# Adaptive Grouping Distributed Compressive Sensing Reconstruction of Plant Hyperspectral Data

**DOI:** 10.3390/s17061322

**Published:** 2017-06-07

**Authors:** Ping Xu, Junfeng Liu, Lingyun Xue, Jingcheng Zhang, Bo Qiu

**Affiliations:** College of Life Information Science & Instrument Engineering, Hangzhou Dianzi University, Hangzhou 310018, China; xuping@hdu.edu.cn (P.X.); fengjliu@126.com (J.L.); qb86880301@126.com (B.Q.)

**Keywords:** hyperspectral image, spectral characteristics of plants, spectral adaptive grouping, compressive sensing

## Abstract

With the development of hyperspectral technology, to establish an effective spectral data compressive reconstruction method that can improve data storage, transmission, and maintaining spectral information is critical for quantitative remote sensing research and application in vegetation. The spectral adaptive grouping distributed compressive sensing (AGDCS) algorithm is proposed, which enables a distributed compressed sensing reconstruction of plant hyperspectral data. The spectral characteristics of hyperspectral data are analyzed and the joint sparse model is constructed. The spectral bands are adaptively grouped and the hyperspectral data are compressed and reconstructed on the basis of grouping. The experimental results showed that, compared with orthogonal matching pursuit (OMP) and gradient projection for sparse reconstruction (GPSR), AGDCS can significantly improve the visual effect of image reconstruction in the spatial domain. The peak signal-to-noise ratio (PSNR) at a low sampling rate (the sampling rate is lower than 0.2) increases by 13.72 dB than OMP and 1.66 dB than GPSR. In the spectral domain, the average normalized root mean square error, the mean absolute percentage error, and the mean absolute error of AGDCS is 35.38%, 31.83%, and 33.33% lower than GPSR, respectively. Additionally, AGDCS can achieve relatively high reconstructed efficiency.

## 1. Introduction

Hyperspectral technology is a breakthrough technology in agriculture remote sensing which enables the dynamic and precise monitoring of crop types and crop growth. Hyperspectral remote sensing technology has been widely used in estimating the yield of crops, agricultural resources surveying, agricultural disaster monitoring, and precision agriculture [1]. Plant quantitative remote sensing technology is widely used in a variety of applications by mining spectral information and setting up spectral retrieving model. For the estimation of crop yield, Nuarrsa et al. extracted a rice area with an overall accuracy of 87.91% using the normalized difference vegetation index (NDVI), radar vegetation index (RVI), and soil-adjusted vegetation index (SAVI) from MODIS time series data [2]. Tornosa et al. assessed the potential of different spectral indices for monitoring rice agricultural practices and hydroperiod dynamics by combining phenometrics and statistical time series approaches [3]. Kang et al. developed spectral indices that can reduce the effects of varied canopy structure and growth stages for the estimation of leaf Chl [4]. Fei et al. optimized the band combinations further, and identified the optimized central bands and suitable bandwidths of the three-band nitrogen planar domain index (NPDI) for estimating the aerial N uptake, N concentration, and above-ground biomass [5]. Atherton et al. linked spectral measurements of fluorescence and the PRI to photosynthesis dynamics at the leaf scale and over short time-scales [6]. Heli et al. found that the values of crop variables may not be accurately determined when they are based solely on the measurements of leaves, especially only the upper leaves, as the values varied greatly among the different vertical leaf and stem layers and the different modules, such as leaves, stems, and spikes [7]. Qiang et al. proposed an iterative method which integrate MODIS, VEGETATION, and MISR data to improve the estimation of leaf area index (LAI) climatology [8]. Mahlein et al. developed specific spectral disease indices (SDIs) for the detection of diseases in crops [9]. Alicia et al. assessed AS1 and AS2 behavior over a cotton crop growing period, testing whether function-fitting procedures can be used to model MODIS AS1 and AS2 and NDVI time series and derive objective AS1 and AS2 phenological metrics that can be used to monitor cotton phenological stages [10]. Veraverbeke et al. evaluated the discriminatory power of existing VIs and thermally-enhanced indices in burned land applications [11]. Jochem et al. introduced an automated spectral band analysis tool (BAT) based on Gaussian process regression (GPR) for the spectral analysis of vegetation properties [12]. Abderrazak et al. proposed a spatiotemporal monitoring method of soil salinization in the Tadla plain in Central Morocco using spectral indices derived from Thematic Mapper (TM) and Operational Land Imager (OLI) data [13]. Ferner et al. tested whether spatio-temporal information on the quality (metabolizable energy content, ME) and quantity (green biomass, BM) of West African forage resources can be correlated to in situ-measured reflectance data [14]. Oz et al. found informative spectral bands in three types of models—vegetation indices (VI), neural network (NN), and partial least squares (PLS) regression—for estimating leaf chlorophyll (Chl) and carotenoid (Car) contents of three unrelated tree species and to assess the accuracy of the models using a minimal number of bands [15]. Jesús et al. proposed a two-step approach to realize simultaneous LAI mapping over green and senescent croplands [16]. Dibyendu et al. demonstrated that total polyphenols of tea can be precisely estimated from a field spectroradiometer at the leaf level irrespective of age of the bushes and farming practices [17].

With the aid of Internet technology, agricultural informatization and agricultural big data have become inevitable trends. In recent years, with the successive launch of hyperspectral satellites and the development of microhyperspectral imagers of UAVs, the applications of hyperspectral remote sensing has become widely available. However, on the other hand, the increase of data volume brings great challenges with respect to data transmission, analysis, and storage [18]. Candes, Donoho, and Tao et al. proposed a new data acquisition and processing theory called compressive sensing (CS) [19,20,21]. Compressive sensing samples data at far below the Nyquist sampling rate by constructing an uncorrelated observation matrix, and the original data is reconstructed by a reconstruction algorithm. It, thus, provides a new way for compressing and reconstructing data with large volume.

At present, many studies have been conducted on applying compressive sensing in processing high-dimensional data. Kang et al. [22] proposed a method of distributed compressive sensing to grouping the video sequences efficiently by studying the correlation of the video sequences. Ly et al. [23] pointed out that the hyperspectral data should be stochastically separated by spectral and spatial partitioning. Chen et al. [24] proposed a sparse method for hyperspectral image target detection. Wang et al. proposed a pixel-based distributed compressive sensing [25], which divides the hyperspectral data into endmember extraction and abundance estimation through a linear mixture model. However, all of these methods did not concern applications of compressive sensing in agriculture, but mainly focused on the spatial reconstruction. 

To promote the application of hyperspectral remote sensing in agriculture, the compressive sensing method provides a new method for the compression and recovery of hyperspectral data. However, at present, the research of compressive sensing is mainly focused on the reconstruction of the spatial image, and the spectral dimension of the hyperspectral data needs to be concerned in information reconstruction. Hyperspectral images have spectral-spatial correlations. The compressive sensing of high-dimensional data using the autocorrelation nature of data to improve the data sparse representation, which is able to reduce the complexity and improve the accuracy of reconstruction. In this study, through the analysis of plant spectral characteristics, a distributed spectral adaptive grouping compressive sensing is proposed and verified.

## 2. Methods

### 2.1. Spectral Adaptive Distributed Compressive Sensing

**Figure 1:**
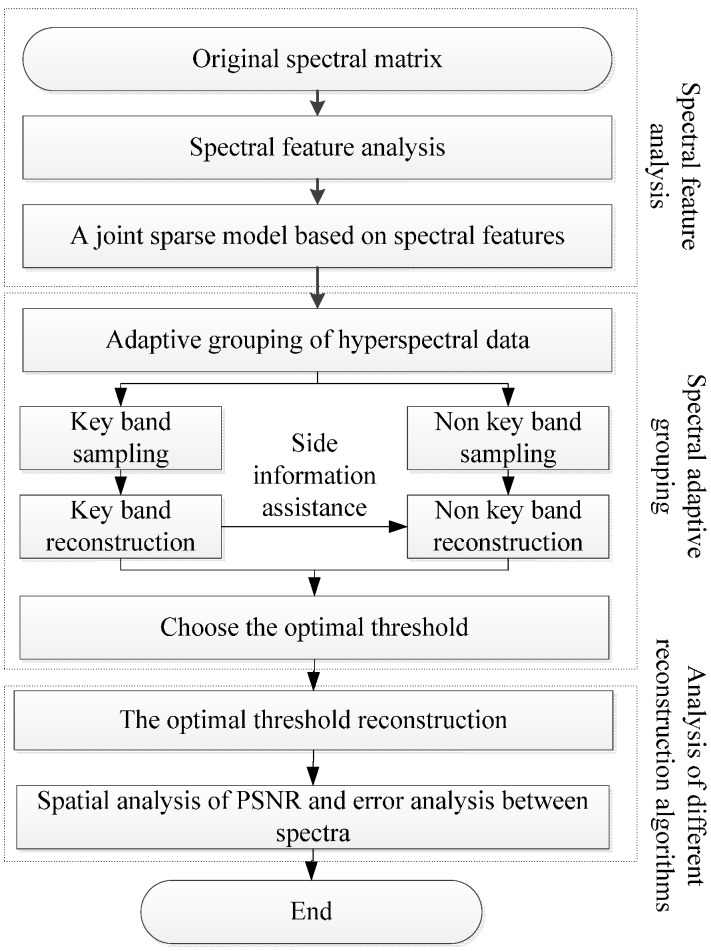
Flowchart of plant hyperspectral compressive sensing reconstruction.

Figure 1 shows the flowchart of the proposed plant hyperspectral compressive sensing reconstruction algorithm. Firstly, the spectral characteristics of hyperspectral data are analyzed and the joint sparse model is constructed. Secondly, the spectral bands are adaptively grouped and the hyperspectral data are compressed and reconstructed on the basis of grouping, so as to determine the optimal grouping threshold. Then, to evaluate the reconstruction effect, AGDCS, OMP [26], and GPSR [27] are used to analyze the PSNR in the space and calculate errors among spectra. 

### 2.2. Analysis of Plant Spectral Characteristics

In this study, a visible and near infrared (VIS-NIR) hyperspectral imaging system covering the spectral wavelengths of 380–1030 nm was used. The system includes a CCD camera (C8484-05, Hamamatsu, Hamamatsu city, Japan), an imaging spectrograph, a lens, two light sources provided by two 150 W quartz tungsten halogen lamps and V10E software (Isuzu Optics Corp., Taiwan) for the computer operating the spectral image system. The spectral resolution is 2.8 nm and the area CCD array detector of the camera has 6,726,512 pixels. The system scans the samples line by line, and the reflected light was dispersed by the spectrograph and captured by the area CCD array detector in spatial-spectral axes. 

**Figure 2:**
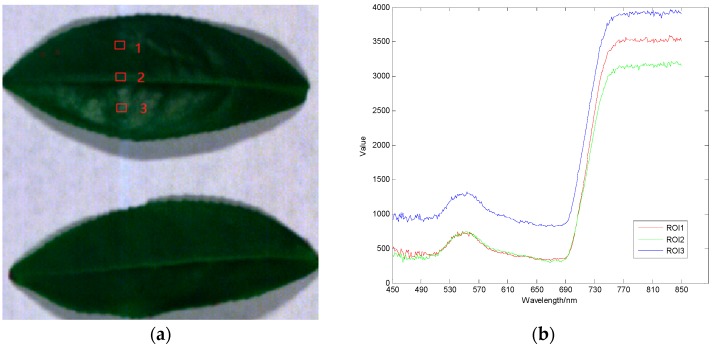
Spatial image of hyperspectral raw data and three regional spectral curves. (**a**) Original data space image; (**b**) The averaged spectrum for each ROI.

The data used in the experiment are hyperspectral images of 12 pieces of *Camellia sinensis*. Data from wavelengths of 450–850 nm and a total of 320 bands of data are chosen to perform the following experiments, and a single pixel is defined by a 12-bit unsigned integer. The images at 661 nm, 553 nm, and 449 nm from the original data are selected as the red, green, and blue channels of the false color composite RGB images, as shown in Figure 2a. Within each leaf, three regions of interest (ROIs) are marked with the size of 3 × 3 pixels. The averaged spectrum for each ROI is demonstrated in Figure 2b.

**Figure 3:**
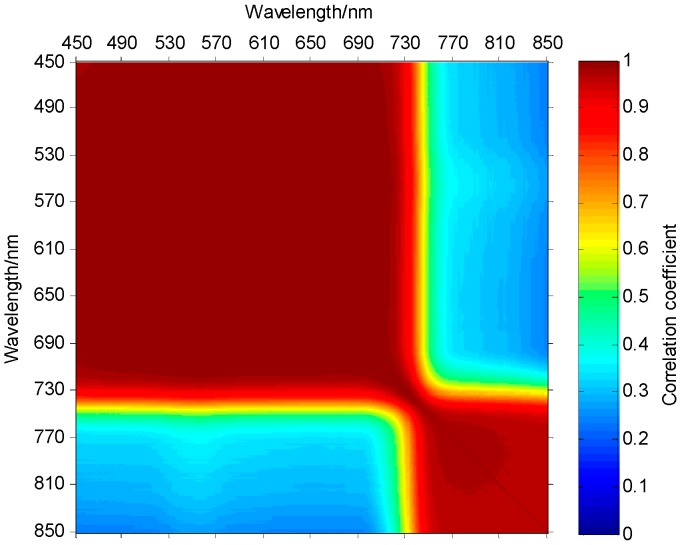
Correlation coefficient diagram.

It can be observed from the spectral curve that despite the averaged spectral curves of different regions are different, the trend of the curves is consistent. The fluctuation of spectral reflectance values in 450–680 nm are relatively small. The spectrum in 680–760 nm rised rapidly. In 760–850 nm, the spectral reflectance is stablized at relatively high values, but the fluctuation within the local adjacent wave bands is relatively large. In order to further study on the distribution of spectral correlation of plant hyperspectral images, the correlation matrix of the hyperspectral bands is shown in Figure 3. It can be observed from the correlation coefficient diagram that the spectral reflectance is high in 450–680 nm and 760–850 nm, and the absolute value of autocorrelation and cross-correlation coefficient between the bands is above 0.9. The correlation between 680–760 nm is small, which is consistent with the variation of the spectral curve. Based on the qualitative analysis of the correlation characteristics of the plant spectrum, the following conclusions are obtained by analyzing the correlation coefficient (r) of each band. 59.41% bands are extremely correlated (|r| > 0.95); 6.93% bands are highly correlated (0.8 < |r| ≤ 0.95); 8.48% bands are moderately correlated (0.5 < |r| ≤ 0.8); whereas 25.18% bands are lowly correlated (0.3 < |r| ≤ 0.5) or not correlated (|r| ≤ 0.3).

### 2.3. Joint Sparse Model

According to the spectral characteristics of the hyperspectral images, there is a general correlation between the bands of the plant hyperspectral images and that more than half of the bands are highly correlated. The higher the correlation between the bands, less difference remained. Based on this, the present study proposed a joint sparse model of bands of plant hyperspectral images [22].

Assuming there are two bands *X_i_* and *X_i+_*_1_, the correlation between *X_i_* and *X_i+_*_1_ can be calculated, where *X_i_* represents the current band. As the result of different wavelength reflection of the same object in the different bands of hyperspectral images, *X_i_* and *X_i+_*_1_ have the same spatial information. Meanwhile, *X_i_* and *X_i+_*_1_ have their own unique spectral information. The *X_i_* and *X_i+_*_1_ can be described as follows:

*X_i_* = *X_c_* + *X*_*i*_*r*_ (1)

*X*_*i*+1_ = *X_c_* + *X*_*i*+1_*r*_ (2)

where *X_c_* is the same part of *X_i_* and *X_i+_*_1_, which refers to the same spatial information, *X_i_r_* and *X_i+_*_1*_r*_ are their own unique parts, which mean the results of different reflections of different wavelengths. *X_i_* is used as a reference to *X_i+_*_1_, and the same spectral estimation (*X_c_*) and the different information error coding (*X_i+_*_1*_r*_) are used as the predictive value of *X_i+_*_1_ in the spectral coding. The joint sparse model can be expressed as below:

*X_i_* = *ΨS_i_* (3)

*X*_*i*+1_*r*_ = *ΨS*_*i*+1_*r*_ (4)

where *S_i_* and *S_i+_*_1*_r*_ are sparse representations of *X_i_* and *X_i+_*_1*_r*_, respectively, and *Ψ* is a canonical orthogonal matrix.

### 2.4. Distributed Compressive Sensing Based on Spectral Characteristics

In the distributed compressive sensing, hyperspectral band data are divided into a series of group of pictures (GOPs) bands. Each GOP consists of several bands which contain one key band and several non-key bands. Then, the sampling rates of the key band and non-key bands are realized by controlling the sampling matrix:

*Y_i_* = Φ*X_i_* = Φ*ΨS_i_* (5)

where Ψ is a canonical orthogonal matrix, *S_i_* is a sparse representation of the original signal in the transform domain, Ψ^H^Ψ = ΨΨ^H^ = *I*, and *I* is a unit matrix. The size of the Φ is *M* × *N*, *M* << *N*, which is the partial block Hadamard matrix [28]. *X_i_* is a band of hyperspectral data, and *Y_i_* is an observed value.

Finally, the GPSR algorithm is used in the key band and the reconstruction of the non-key band is assisted by using the information from the key band. Compressive sensing reconstruction is the process of solving Equation (6). The solution of *l*_0_ norm is an NP-hard problem. The minimization problem of *l*_1_ norm is equivalent to that of *l*_0_ norm under certain conditions [29], so Equation (6) can be transformed to Equation (7). In this paper, GPSR and OMP are chosen to reconstruct *X_i_* on the basis of the *Y_i_*:

(6) $$\overset{\wedge }{x}=\arg\;\min{\left\|x\right\|}_{0}\;\mathrm{st}\;\Phi x=y$$

(7) $$\overset{\wedge }{x}=\arg\;\min{\left\|x\right\|}_{1}\;\mathrm{st}\;\Phi x=y$$

### 2.5. Spectral Adaptive Grouping and Selection of Key Bands

Based on the above analysis, non-key bands can be reconstructed by the key band with side information assist. Therefore, it is very important to effectively group bands and select more effective key bands. In this study, PSNR is used as a basis for adaptive grouping of hyperspectral data and selecting key bands. The steps of adaptive grouping algorithm are as follows:

- Step 1: Solve all PSNRs between the first band and each of the rest of the bands, and those in the rest of the bands and those whose PSNRs are greater than the threshold are all selected and classified into the group of the first band.
- Step 2: Set up a new set from the remaining bands and repeat Step 1 to construct a new group.
- Step 3: Repeat Step 2 until all bands are assigned to different groups.

In the adaptive band grouping algorithm, we can see that in every grouping, the PSNR values of the first band and other bands are greater than the threshold value. Given that the first band in the group has high similarity with the remaining bands, the first band of each group is determined as the key band and the other bands are non-key bands.

### 2.6. Error Evaluation Methods

The mean absolute percentage error (*MAPE*), the mean absolute error (*MAE*), and the root mean square error (*RMSE*) are used to evaluate the reconstructed effectiveness.

*MAPE*, also known as mean absolute percentage deviation (*MAPD*), is a measure of prediction accuracy of a forecasting method in statistics, and is defined by the formula:

(8) $$MAPE=\frac{\sum_{t=1}^{n}\left|\frac{A_{t}-F_{t}}{A_{t}}\right|}{n}$$

*MAE* is a quantity used to measure how close forecasts or predictions are to the eventual outcomes. The MAE is given by:

(9) $$MAE=\frac{\sum_{i=1}^{n}\left|y_{i}-x_{i}\right|}{n}$$

*RMSE* represents the sample standard deviation of the differences between predicted values and observed values. The *RMSE* is given by:

(10) $$RMSE=\sqrt{\frac{\sum_{t=1}^{n}{\left({\overset{⌢}{y}}_{t}-y_{t}\right)}^{2}}{n}}$$

## 3. Results and Discussion

In the experiments, the software platform is MATLAB R2012a and the hardware platform is a Lenovo notebook computer in which the CPU is an Intel I3-2350M clocked at 2.3 GHz with 6 GB of memory.

### 3.1. The Results of Spectral Adaptive Threshold Grouping

#### 3.1.1. The Results of Adaptive Grouping and the Different Sampling Rate of Key and Non-Key Bands

Key bands are sampled at a high rate and all sampling rates are from 0.1 bpp (bits per pixel) to 0.5 bpp. In case the sampling rate of the GPSR algorithm is over 0.4 bpp, the PSNR value changes slowly. The sampling rate of the key band is set to 0.5 bpp. The grouping results of non-key bands at different thresholds and the sampling rates of non-key bands are shown in Table 1.

It can be seen from Table 1, with the increase of the number of groupings, that the sampling rate of the non-key band decreases gradually, especially at a low sampling rate. When the threshold is set to 30 dB and the overall sampling rate is 0.1 bpp, the number of groupings will reach 65 and the sampling rate of the non-key band will be negative.

#### 3.1.2. Analysis of Results of Adaptive Band Grouping Reconstruction

**Figure 4:**
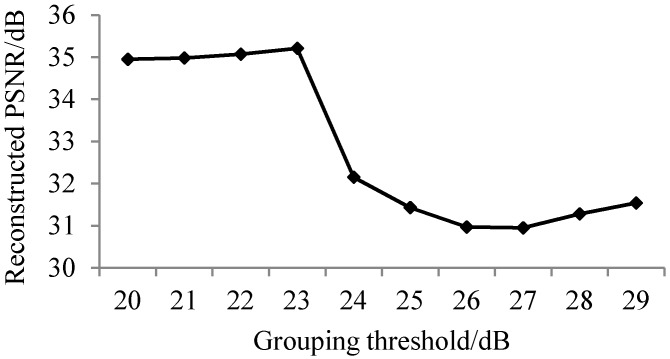
Average reconstructed PSNR of key bands for different groups.

**Figure 5:**
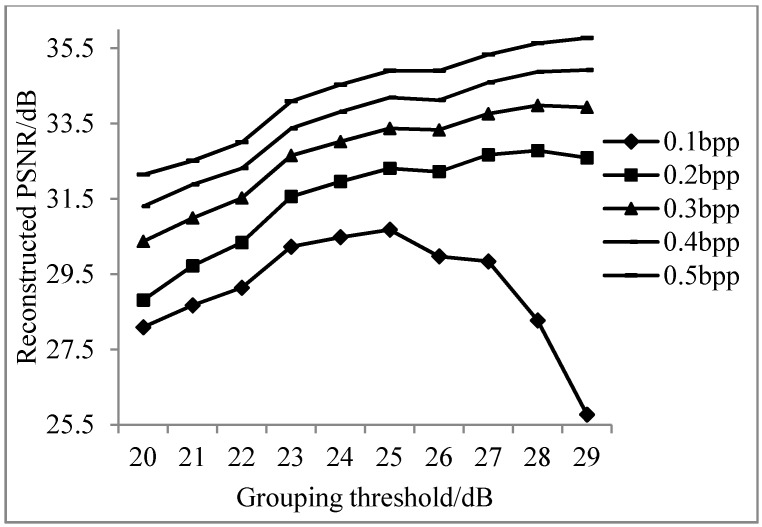
Average reconstructed PSNR of non-key bands for different groups.

**Figure 6:**
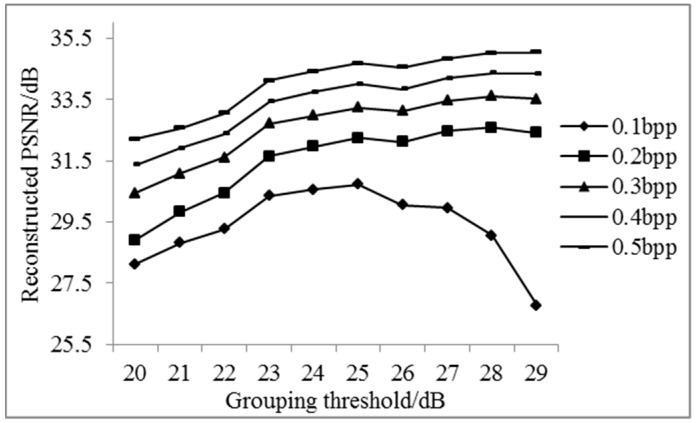
Average reconstructed PSNR of all bands for different groups.

According to the sampling rate of the non-key bands calculated in Table 1, experimental results of the reconstructed PSNR of key bands, non-key bands, and all bands are shown as Figure 4, Figure 5 and Figure 6.

**Figure 7:**
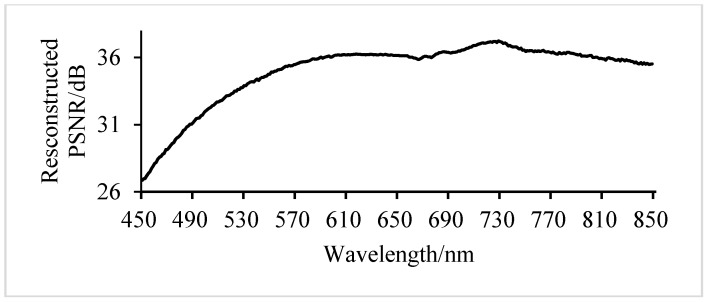
Reconstructed PSNR of each band at a sampling rate of 0.5 bpp.

It can be seen from Figure 4 that the PSNR values of different groupings of key band reconstruction vary significantly, as the sampling rate of key bands is set to 0.5 bpp. To explain this phenomenon, more experiments are implemented for each band at a sampling rate of 0.5 bpp, as shown in Figure 7.

In Figure 7, when the sampling rate is 0.5 bpp, the reconstructed PSNR values of different bands are quite different. According to the spectral characteristic curve, the difference of the spectrum with a proximate reconstructed PSNR value is insignificant. The lower the PSNR threshold is, the more groups will emerge and the higher the average PSNR value of key bands will be, and vice versa. 

As can be seen from Figure 5 and Figure 6, when the sampling rate is larger, the reconstructed PSNR values of non-key bands and all bands are both higher. More groupings indicate less efficiency of the overall reconstruction. In order to obtain good reconstructed PSNR for all sampling rates from 0.1 bpp to 0.5 bpp, the grouping PNSR threshold of 25 dB is chosen in the following experiments.

### 3.2. Spatial Domain Reconstruction Analysis

**Figure 8:**
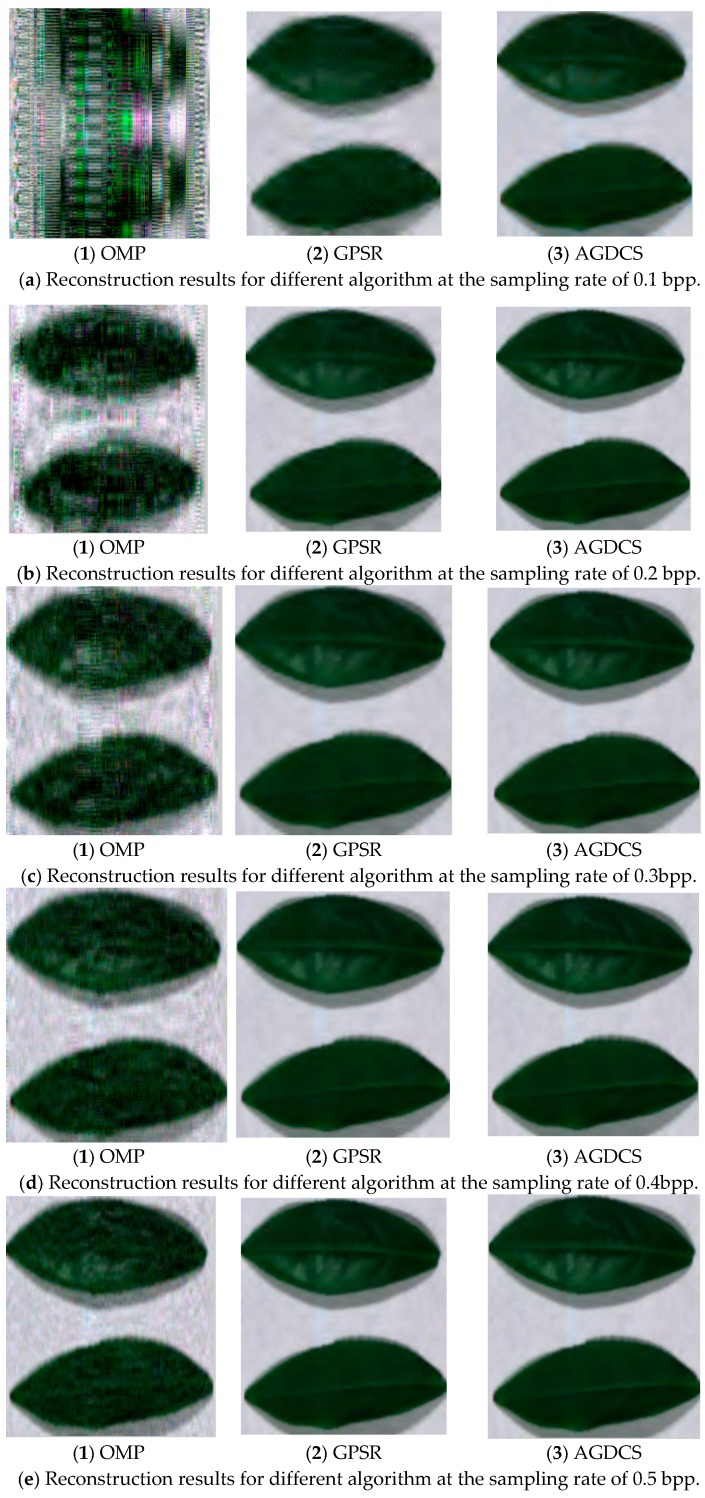
Comparison of reconstruction results for different algorithms at different sampling rates.

Twenty-five decibels was selected as the overall threshold for further analysis using OMP and GPSR algorithms. The reconstructed results are analyzed using the subjective evaluation and average peak signal to noise ratio in the spatial domain. To facilitate a visual comparison, 661 nm, 553 nm, and 449 nm are selected, respectively, as red, green, and blue channels to form the synthesized RGB image. Figure 8 illustrates the experimental results for OMP, GPSR, and AGDCS under the sampling rate from 0.1 bpp to 0.5 bpp. The fidelity of reconstructed images of all algorithms are significantly related with the sampling rate, especially at low sampling rates. The increasing of the sampling rate can significantly improve the subjective quality of the reconstructed image. Better subjective quality can be obtained using AGDCS compared with the other algorithms. The reconstructed subjective quality of AGDCS is very close to that of GPSR and significantly better than that of OMP at high sampling rates (great than or equal to 0.4 bpp). Experimental results show that the side information assists of key bands can improve the quality of reconstruction at low sampling rates. 

**Figure 9:**
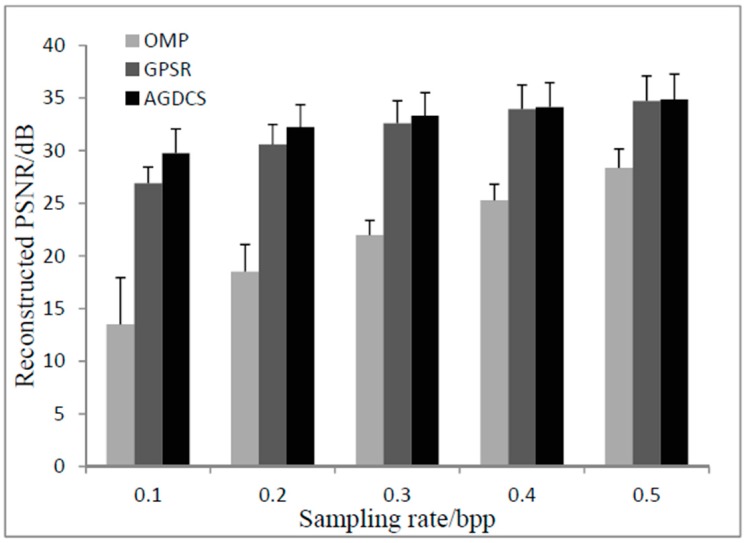
Comparison of the reconstructed PSNR with different reconstruction methods.

It can be seen from Figure 9 that the reconstructed PSNRs of AGDCS and GPSR are significantly higher than that of OMP under sampling rates from 0.1 bpp to 0.5 bpp. At low sampling rates from 0.1 bpp to 0.3 bpp, the reconstructed PSNR of AGDCS is significantly higher than that of GPSR. At high sampling rates (greater than or equal to 0.4 bpp), the reconstructed PSNR of AGDCS approaches that of GPSR. It is shown that the joint sparse model improves the reconstructed PSNR in the spatial domain, especially at low sampling rates. Additionally, from the error bar of Figure 9, the standard deviation of AGDCS is close to that of GPSR. 

### 3.3. Spectral Domain Reconstruction Results Analysis

The spectral analysis on vegetation is an important basis for the monitoring of crop growing status and crop stressors in precision farming. According to regions of interest (ROIs) in Figure 1, the average reconstructed spectral curves of ROIs using OMP, GPSR, and AGDCS are calculated at different sampling rates, from 0.1 bpp to 0.5 bpp, respectively.

**Figure 10:**
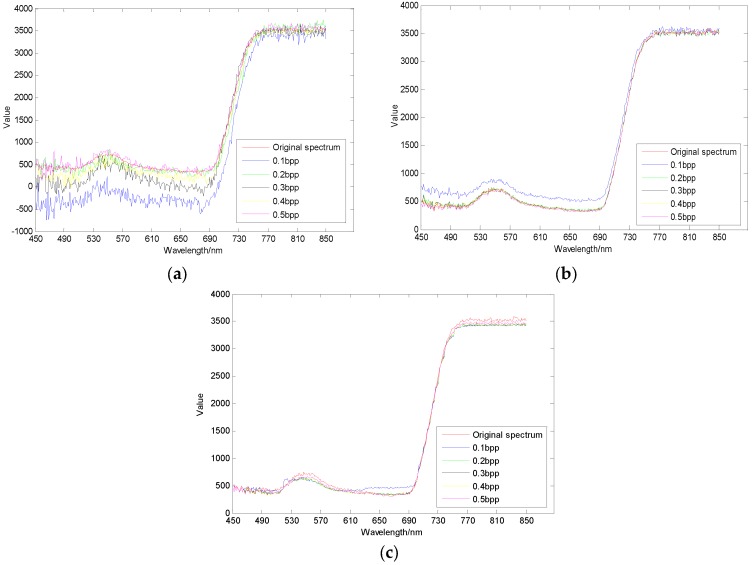
Comparison of reconstructed spectral curves of ROI1. (**a**) OMP; (**b**) GPSR; (**c**) AGDCS.

**Figure 11:**
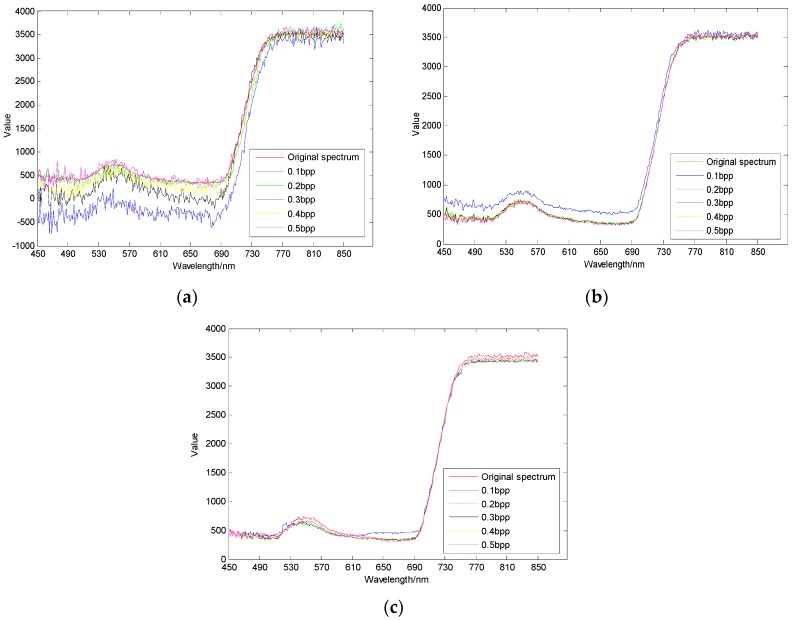
Comparison of reconstructed spectral curves of ROI2. (**a**) OMP; (**b**) GPSR; (**c**) AGDCS.

**Figure 12:**
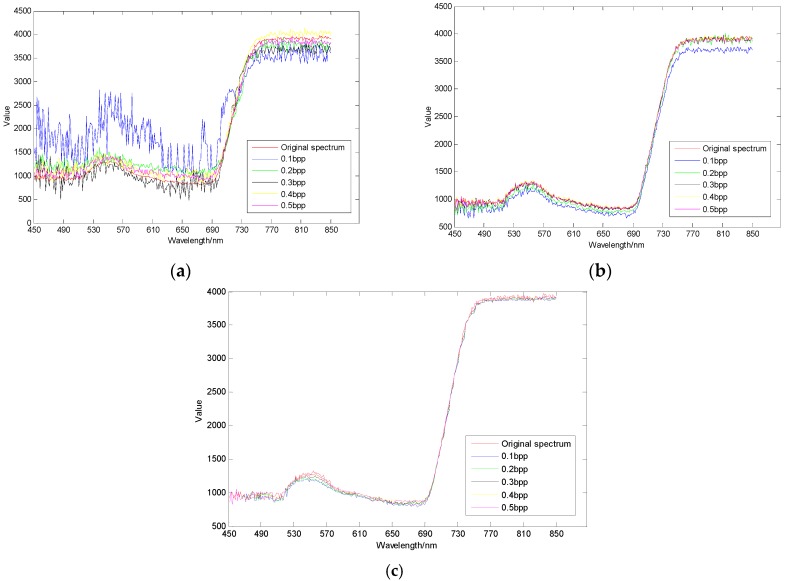
Comparison of reconstructed spectral curves of ROI3. (**a**) OMP; (**b**) GPSR; (**c**) AGDCS.

As shown in Figure 10, Figure 11 and Figure 12, different regions have different sensitivity to compressive sensing reconstruction. There is a significant correlation between the reconstructed algorithms and the sampling rates, and the reconstructed effect is improved with the increase of the sampling rate. Additionally, the reconstructed curves of AGDCS are significantly better than those of the others for ROI2 and ROI3, and that of OMP is the lowest under different sampling rates for these three ROIs. For ROI1, part of reconstructed curves of AGDCS is worse than that of GPSR. The reconstructed effectiveness of the various methods is quantified by introducing MAPE, MAE, and RMSE as shown in Table 2. MAPE, MAE, and RMSE of AGDCS are better than those of the others when the sampling rate is less than 0.2 bpp. MAPE, MAE, and RMSE of AGDCS is comparable to those of GPSR when the sampling rate of greater than or equal to 0.3 bpp, and those of OMP are lower than those of the others.

### 3.4. Results of Spectral Indices of Physiological Properties

As it can been observed from Table 3, Table 4, Table 5 and Table 6, *RMSE* comparisons of the spectral indices of CRI550, CRI700, Dep550–750, and Area550–750, of different methods for three ROIs at different sampling rates are given. For spectral indices of CRI550, CRI700, Dep550–750, and Area550–750 for three ROIs at different sampling rates, the *RMSE* of OMP is relatively higher than that of the other methods. *RMSE*s of these four spectral indices of AGDCS are almost all less than those of GPSR for ROI2 and ROI3 at different sampling rates. For ROI1, the situation is different. The *RMSE*s of these four spectral indices of GPSR are sometimes less than those of AGDCS. These results are just like Figure 10 shows and, although part of the results for AGDCS are worse than GPSR, the overall results of AGDCS are better than GPSR.

### 3.5. Results of Average Reconstructed Time

**Figure 13:**
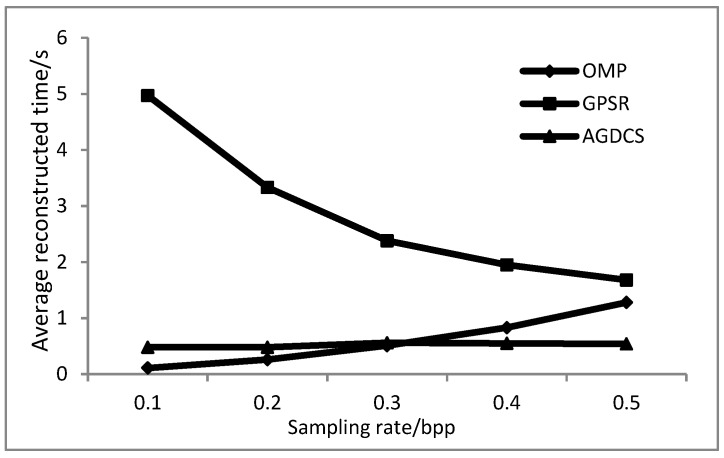
Comparison of the average reconstructed time of all bands for different algorithms.

The experimental results of average reconstructed time of all bands for different algorithms are shown in Figure 13. The reconstruction efficiency of the OMP algorithm is positively correlated with the sampling rate, while the reconstruction efficiency of the GPSR algorithm is negatively correlated with the sampling rate. The average reconstruction time of AGDCS is relatively stable. The average reconstruction time of GPSR is much higher than that of the others. As a result, AGDCS exhibited relatively high reconstructed efficiency.

### 3.6. Discussion

The OMP is a typical greedy algorithm whose reconstructed effect is worse than that of GPSR, which is a type of convex optimization algorithm. Thus, GPRS is chosen as the reconstructed algorithm for AGDCS to reconstruct plant hyperspectral data. AGDCS is based on the joint sparse model of plant hyperspectral images which can make full use of high inter-spectral correlation and the key bands to assist reconstruction of hyperspectral data. Unlike the AGDCS, GPSR does not take inter-spectral correlation into account, but only uses intra-spectral correlation to process hyperspectral data band by band. Additionally, the grouping strategy of AGDCS is to put those bands whose PSNR values are close to its key band into a group in which there is only one key band so the same spatial information of one group provides the primary information and different information errors become relatively low. However, GPSR processes each band as a key band. It leads to the reconstructed performance of AGDCS being quite good. This explains the phenomenon that the reconstructed performance of AGDCS is better than that of GPSR. For AGDCS, the reconstruction of the non-key bands plays the main role, whose main computational burden is to process the different information errors between the key band and the non-key band, which is significantly lower than that of the key-band. Thus, the reconstructed efficiency of AGDCE is extraordinarily higher than that of GPSR.

## 4. Conclusions

There is a high spectral correlation for plant hyperspectral data. The joint sparse model based on spectral characteristics can not only improve the fidelity of reconstructed plant hyperspectral images, but also effectively reduces the reconstructed error in spectral domain more efficiently. Under a relatively low sampling rate (less than 0.2 bpp), the PSNR of AGDCS is 13.72 dB higher than that of the OMP algorithm, and is 1.66 dB higher than that of the GPSR algorithm. For the errors in the spectral domain, the average normalized root mean square error, the mean absolute percentage error, and the mean absolute error of AGDCS decrease by 35.38%, 31.83%, and 33.33% than those of the GPSR algorithm, respectively. Additionally, AGDCS can achieve a relatively high reconstructed efficiency. 

More extensive research can be studied in the future. For example, new compressive sensing algorithms can be involved in denoising the plant hyperspectral data. A high-performance reconstructed method should be proposed to improve the reconstructed efficiency.

## Figures and Tables

**Table 1:** Sampling rates of non-key bands in different groups.

Threshold/dB	Groups	Sampling Rate/bpp				
		0.1	0.2	0.3	0.4	0.5
20	6	0.092	0.194	0.296	0.398	0.500
21	7	0.091	0.193	0.296	0.398	0.500
22	7	0.091	0.193	0.296	0.398	0.500
23	8	0.090	0.192	0.295	0.397	0.500
24	15	0.080	0.185	0.290	0.395	0.500
25	21	0.072	0.179	0.286	0.393	0.500
26	29	0.060	0.170	0.280	0.390	0.500
27	36	0.049	0.162	0.275	0.387	0.500
28	45	0.035	0.151	0.267	0.384	0.500
29	56	0.015	0.136	0.258	0.379	0.500

**Table 2:** Error analysis.

Error Analysis of Different Algorithms		Sampling Rate/bpp				
		0.1	0.2	0.3	0.4	0.5
*MAPE*	OMP	0.9683	0.5158	0.2861	0.2105	0.1598
	GPSR	0.1560	0.1006	0.0796	0.0689	0.0617
	AGDCS	0.1005	0.0814	0.0728	0.0680	0.0639
*MAE*	OMP	0.4599	0.2408	0.1434	0.1035	0.0787
	GPSR	0.0798	0.0529	0.0424	0.0369	0.0329
	AGDCS	0.0544	0.0451	0.0401	0.0370	0.0345
*RMSE*	OMP	0.5722	0.3003	0.1803	0.1303	0.0994
	GPSR	0.1065	0.0698	0.0556	0.0481	0.0428
	AGDCS	0.0710	0.0580	0.0519	0.0479	0.0447

**Table 3:** RMSE comparison of spectral indices of CRI550.

Index Analysis of Different Algorithms		Sampling Rate/bpp				
		0.1	0.2	0.3	0.4	0.5
ROI1	OMP	15.196	1.216	24.319	10.768	1.027
	GPSR	0.499	0.209	0.219	0.187	0.267
	AGDCS	0.262	0.623	0.514	0.495	0.377
ROI2	OMP	1.683	7.504	0.773	81.952	0.778
	GPSR	0.628	0.481	0.372	0.359	0.292
	AGDCS	0.535	0.410	0.345	0.375	0.432
ROI3	OMP	0.623	1.116	1.400	0.707	0.538
	GPSR	0.536	0.225	0.206	0.209	0.200
	AGDCS	0.259	0.225	0.221	0.209	0.232

**Table 4:** RMSE comparison of spectral indices of CRI700.

Index Analysis of Different Algorithms		Sampling Rate/bpp				
		0.1	0.2	0.3	0.4	0.5
ROI1	OMP	103.698	1.246	28.733	13.530	1.468
	GPSR	0.543	0.266	0.250	0.220	0.279
	AGDCS	0.2367	0.727	0.584	0.538	0.395
ROI2	OMP	2.054	164.593	0.956	96.027	1.340
	GPSR	0.601	0.424	0.315	0.312	0.266
	AGDCS	0.513	0.405	0.333	0.350	0.403
ROI3	OMP	0.691	0.950	2.181	0.728	0.557
	GPSR	0.667	0.314	0.245	0.270	0.273
	AGDCS	0.325	0.317	0.285	0.314	0.327

**Table 5:** RMSE comparison of spectral indices of Dep550–750.

Index Analysis of Different Algorithms		Sampling Rate/bpp				
		0.1	0.2	0.3	0.4	0.5
ROI1	OMP	0.403	0.084	0.210	0.116	0.065
	GPSR	0.075	0.017	0.022	0.015	0.009
	AGDCS	0.032	0.008	0.006	0.011	0.009
ROI2	OMP	0.092	0.291	0.085	0.095	0.043
	GPSR	0.080	0.049	0.037	0.032	0.025
	AGDCS	0.057	0.037	0.029	0.027	0.024
ROI3	OMP	0.103	0.083	0.166	0.052	0.044
	GPSR	0.064	0.035	0.024	0.024	0.024
	AGDCS	0.032	0.022	0.027	0.027	0.022

**Table 6:** RMSE comparison of spectral indices of Area550–750.

Index Analysis of Different Algorithms		Sampling Rate/bpp				
		0.1	0.2	0.3	0.4	0.5
ROI1	OMP	0.362	0.061	0.106	0.078	0.059
	GPSR	0.093	0.013	0.022	0.019	0.018
	AGDCS	0.014	0.031	0.026	0.030	0.026
ROI2	OMP	0.244	0.258	0.084	0.083	0.125
	GPSR	0.114	0.073	0.053	0.044	0.036
	AGDCS	0.056	0.037	0.037	0.033	0.033
ROI3	OMP	0.456	0.171	0.095	0.132	0.099
	GPSR	0.112	0.064	0.039	0.031	0.035
	AGDCS	0.051	0.036	0.033	0.027	0.028
